# Headwater Capture Evidenced by Paleo-Rivers Reconstruction and Population Genetic Structure of the Armored Catfish (*Pareiorhaphis garbei*) in the Serra do Mar Mountains of Southeastern Brazil

**DOI:** 10.3389/fgene.2017.00199

**Published:** 2017-12-05

**Authors:** Sergio M. Q. Lima, Waldir M. Berbel-Filho, Thais F. P. Araújo, Henrique Lazzarotto, Andrey Tatarenkov, John C. Avise

**Affiliations:** ^1^Laboratório de Ictiologia Sistemática e Evolutiva, Departamento de Botânica e Zoologia, Centro de Biociências, Universidade Federal do Rio Grande do Norte, Natal, Brazil; ^2^Department of Ecology and Evolutionary Biology, University of California, Irvine, Irvine, CA, United States; ^3^Department of Biosciences, College of Science, Swansea University, Swansea, United Kingdom; ^4^Laboratório de Ecologia de Peixes, Departamento de Ecologia, Instituto de Biologia, Universidade Federal do Rio de Janeiro, Rio de Janeiro, Brazil; ^5^California Academy of Sciences, San Francisco, CA, United States

**Keywords:** biogeography, Atlantic Forest, phylogeography, conservation genetics, Loricariidae

## Abstract

Paleo-drainage connections and headwater stream-captures are two main historical processes shaping the distribution of strictly freshwater fishes. Recently, bathymetric-based methods of paleo-drainage reconstruction have opened new possibilities to investigate how these processes have shaped the genetic structure of freshwater organisms. In this context, the present study used paleo-drainage reconstructions and single-locus cluster delimitation analyses to examine genetic structure on the whole distribution of *Pareiorhaphis garbei*, a ‘near threatened’ armored catfish from the Fluminense freshwater ecoregion in Southeastern Brazil. Sequences of two mitochondrial genes (cytochrome b and cytochrome c oxidase subunit 1) were obtained from five sampling sites in four coastal drainages: Macaé (KAE), São João (SJO), Guapi-Macacu [sub-basins Guapiaçu (GAC) and Guapimirim (GMI)], and Santo Aleixo (SAL). Pronounced genetic structure was found, involving 10 haplotypes for *cytB* and 6 for *coi*, with no haplotypes shared between localities. Coalescent-based delineation methods as well as distance-based methods revealed genetic clusters corresponding to each sample site. Paleo-drainage reconstructions showed two putative paleo-rivers: an eastern one connecting KAE and SJO; and a western one merging in the Guanabara Bay (GAC, GMI, and SAL). A disagreement was uncovered between the inferred past riverine connections and current population genetic structure. Although KAE and SJO belong to the same paleo-river, the latter is more closely related to specimens from the Guanabara paleo-river. This discordance between paleo-drainage connections and phylogenetic structure may indicate an ancient stream-capture event in headwaters of this region. Furthermore, all analyses showed high divergence between KAE and the other lineages, suggesting at least one cryptic species in the latter, and that the nominal species should be restricted to the Macaé river basin, its type locality. In this drainage, impacts such as the invasive species and habitat loss can be especially threatening for such species with a narrow range. Our results also suggest that freshwater fishes from headwaters in the Serra do Mar mountains might have different biogeographical patterns than those from the lowlands, indicating a complex and dynamic climatic and geomorphological history.

## Introduction

Two alternative hypotheses are typically invoked to explain disjoint distributions encompassing more than one drainage basin of strictly freshwater species: past anastomoses of coastal basins due to marine regression episodes that resulted in paleo-drainage connections or headwater stream-captures caused by tectonic adjustments (Albert and Reis, 2011). Stream capture occurs when a river changes its course and connects with another drainage system as a result of geomorphological changes (Bishop, 1995; Wilkinson et al., 2006), whereas paleo-drainage connections happen when sea levels are lowered by climatic changes, causing basins that previously were isolated to coalesce (Voris, 2000; Dias et al., 2014). To assess how these processes have influenced the genetic structure of freshwater fishes, recently developed GIS-based methods have been used to reconstruct paleo-river systems (Dias et al., 2014; Thomaz et al., 2015, 2017). These methods use topographical and bathymetrical data to reconstruct land exposure, fine-scale depth and steepness of exposed areas (which represent putative riverbeds), as well as putative flow and basin limits (Thomaz et al., 2015). Bathymetric-based reconstructions proved to be highly concordant with the available geological record for paleo-drainages (Dias et al., 2014), supporting GIS-based reconstruction as a powerful and reliable tool for paleo-drainage reconstructions, especially in regions with scarce geological data (Thomaz et al., 2015).

Headwater regions usually exhibit high levels of endemism and microhabitat specialization (Albert et al., 2011; Buckup, 2011). One such example of headwater taxa involves loricariid catfishes of the genus *Pareiorhaphis*. These rheophilic (fast-water) fishes usually show a narrow distribution, with 20 of 25 described species (80%) restricted to a single basin (Pereira et al., 2017). One of the exceptions within the genus is *Pareiorhaphis garbei* (Ihering, 1911), a species found in four coastal basins of the Rio de Janeiro State in southeastern Brazil: Macaé, São João, Guapi-Macacu, and Santo Aleixo drainages (Maia et al., 2013). This armored catfish is restricted to clear-water streams with fast-flowing high-oxygen waters and predominance of rocky (bouldered) substrate (Lazzarotto et al., 2007), and these habitats only occur in the headwaters or upper reaches of coastal basins (Maia et al., 2013). The geographic distribution of *P. garbei* lies entirely within the Atlantic Forest biodiversity hotspot (Myers et al., 2000), which in turn is situated within the Fluminense freshwater ecoregion, a small area comprising about 110 freshwater fish species and a high proportion (42%) of endemic species (Albert et al., 2011).

Due to the intense modification of natural areas by rural and urban expansion, many freshwater fish species in the Fluminense ecoregion are threatened. Previously, *P. garbei* was classified as an ‘endangered’ species [Ministério do Meio Ambiente [MMA], 2004], but it is currently considered ‘near threatened’ and is, thus, a priority species for research [Instituto Chico Mendes de Conservação da Biodiversidade [ICMBio], 2014]. Deforestation, chemical pesticides, and the introduction of rainbow trout *Oncorhynchus mykiss* (Walbaum, 1792) were shown to be threats for *P. garbei* (Pereira and Brito, 2008), even though most records for the species are in protected areas (Maia et al., 2013). However, molecular studies indicate that some taxa putatively distributed along coastal basins of the Fluminense ecoregion may be species complexes with individually narrow distributions (Villa-Verde et al., 2012; Cherobim et al., 2016; Roxo et al., 2017).

Application of molecular phylogenetic analyses and paleo-drainage reconstruction of headwater taxa might reveal their population histories, including information relevant for taxonomy and conservation. Single-locus cluster delineation, often based on mitochondrial DNA, is drastically changing investigations of global biodiversity (Hajibabaei et al., 2007; Pereira L.H. et al., 2013). Clusters revealed by this analysis are considered operational taxonomic units (OTUs). Units that are supported by several clustering methods with different assumptions offer a starting point for taxonomic reevaluation (Kekkonen and Hebert, 2014). This study addresses the genetic structure of *P. garbei* to infer historical processes that may have influenced the species’ disjointed distribution along coastal basins of the Serra do Mar. In particular, we will address possible paleo-drainage connections and headwater captures. If the lineage patterns are consistent with reconstructed paleo-rivers, then anastomosis of coastal basins by regressive glacial episodes would likely explain the current distribution. However, if the observed phylogeographic pattern is incongruent with the paleo-river reconstruction, then stream capture would likely explain lineage patterns along the headwaters of distinct basins. Furthermore, the species delimitation analyses will provide complementary data for systematic studies, as well as useful information for evaluating the conservation status of *P. garbei*.

## Materials and Methods

### Sampling

**FIGURE 1:**
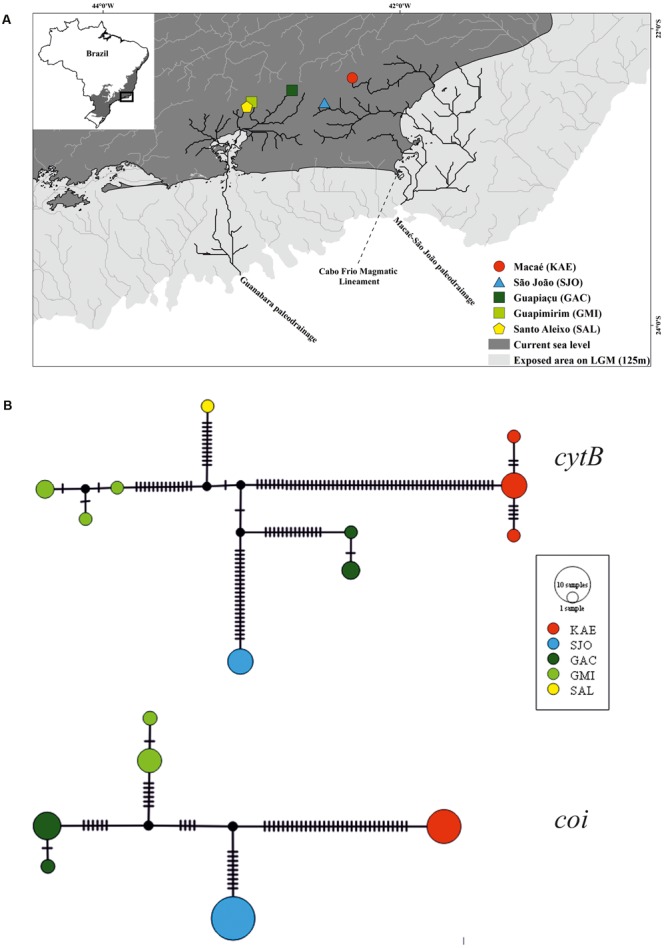
**(A)** Paleo-drainages reconstruction of coastal basins from Serra do Mar mountains, Rio de Janeiro, southeastern Brazil. Dark gray represents the continent at current sea level and light gray a putative exposure of the continental shelf during a sea regression of 125 m. The Atlantic Forest distribution is represented in grey on the inset map. **(B)** Haplotypes networks of cytochrome b (*cytB*) and cytochrome oxidase I (*coi*) in *Pareiorhaphis garbei*.

Specimens of *P. garbei* were captured in five localities of four hydrographic basins covering the entire known species distribution (Maia et al., 2013): Macaé (KAE), São João (SJO), Guapi-Macacu [sub-basins Guapiaçu (GAC) and Guapimirim (GMI)], and Santo Aleixo (SAL) (**Table 1**). Whereas the rivers Santo Aleixo and Guapi-Macacu flow into the Guanabara Bay in the same mangrove area (and their river mouths are less than 2 km apart), the São João and Macaé rivers flow to the east coast of Rio de Janeiro State directly into the Atlantic Ocean, about 100 km from Guanabara Bay (Costa, 2014) (**Figure 1**). Muscle tissues or fin clips were obtained from specimens preserved in absolute ethanol. These individuals are stored at the ichthyological collections of the Universidade Federal do Rio Grande do Norte and of the Departamento de Ecologia of the Universidade Federal do Rio de Janeiro (**Table 1**).

**Table 1:** Sampling sites of *Pareiorhaphis garbei* in the Serra do Mar coastal basins, Rio de Janeiro State, southeastern Brazil.

Locality	Basin	Geographic Coordinates	Altitude (m)	CU	Vouchers (UFRN)	*cytB*	*coi*
Macaé (KAE)	Macaé	22°25′50.1″ S 42°32′20.2″ W	1090	1	098, 108	6	6
São João (SJO)	São João	22°29′54.5″ S 42°30′34.6″ W	90	2	010, 1025	4	10
Guapiaçu (GAC)	Guapi-Macacu	22°24′53″ S 42°43′37″ W	180	3	DEPR J8483	3	5
Guapimirim (GMI)	Guapi-Macacu	22°29′32.1″S 43°00′03.3″ W	400	3	092	4	4
Santo Aleixo (SAL)	Santo Aleixo	22°31′29.7″ S 43°01′54.1″ W	270	3	103, 111	1	–

*Conservation Units (CU) are as follows: (1) Três Picos State Park, (2) Bacia do Rio São João Environmental Protection Area, and (3) Petrópolis Environmental Protection Area/Serra dos Órgãos National Park. Vouchers: (UFRN) Universidade Federal do Rio Grande do Norte fish collection, (DEPRJ) Departamento de Ecologia Universidade Federal do Rio de Janeiro fish collection. Columns *cytB* and *coi* show number of specimens studied at each gene.*

### Paleo-Drainages Reconstruction

To access paleo-drainages connections during the Last Glacial Maximum (LGM), topographical and bathymetrical data were retrieved from the digital elevation model at 30 arc-second resolution^1^. This layer was uploaded into ArcGIS10 software, and the *Hydrological* tools add-in was used to reconstruct the paleo-drainages, following the steps described in Thomaz et al. (2015). First, the area exposed during the LGM (-125 m) was identified using the *Contour* followed by *Mask*. Afterwards, the tools *Fill, Flow Direction*, and *Basin* were used, respectively, to fill depressions on the surface, identify the steepness within each cell, and define the basin borders. Finally, the *Stream order* function was used to estimate putative paleo-rivers.

### DNA Extraction and Amplification

Genomic DNA samples were extracted using phenol/chloroform/isopropanol with ethanol precipitation protocol (Milligan, 1998), and mitochondrial DNA fragments of genes encoding cytochrome oxidase subunit I (*coi*) and cytochrome b (*cytB*) were amplified and sequenced using primers and conditions proposed by Villa-Verde et al. (2012). Overall, 18 and 25 specimens were amplified and sequenced for *cytB* (1056 bp) and *coi* (878 bp), respectively (GenBank MG251217-251259) (**Table 1**).

### Phylogenetic Analysis and Species Delimitation

Sequences were edited in MEGA6 (Tamura et al., 2013), then aligned using the MUSCLE algorithm (Edgar, 2004). *P*-distance (*cytB*) and Kimura-2-parameter (K2P) (*coi*) distances for markers were also calculated in MEGA6. A Bayesian phylogenetic reconstruction for both *cytB* and *coi* data was done in BEAST v. 1.7 (Drummond et al., 2012) using HKY+G (*cytB*) and K2P+G (*coi*) as nucleotide substitution models, as suggested by jModeltest 2 (Darriba et al., 2012). An uncorrelated relaxed lognormal model with estimated rate was used, with ucld.mean parameter set and uniform distribution (0 and 10 as lower and upper boundaries). Remaining parameters were set as default. The length of the MCMC chain was 10,000,000 runs with sampling every 1000 runs. ESS (> 200) values were checked using Tracer v. 1.5 (Rambaut, 2009). The initial 2000 trees were discarded as burn-in period, and a final tree was reconstructed using TreeAnnotator v.1.50. Each haplotype network was inferred using the TCS method on PopART (Leigh and Bryant, 2015). *Pareiorhaphis* cf. *bahianus* from Contas river basin in Bahia State was used as out-group in an additional Bayesian phylogeny, using the same parameters described above, for each gene to corroborate the relationships among *P. garbei* lineages (**Supplementary Figure S1**).

For increased robustness in delimiting OTUs, five single-locus species-delimitation analyses were performed using both markers separately: single-threshold of Generalized Mixed Yule-Coalescent (sGMYC) (Fujisawa and Barraclough, 2013); multiple-threshold GMYC (mGMYC) (Fujisawa and Barraclough, 2013); Bayesian implementation of Poisson Tree Process (bPTP) (Zhang et al., 2013); multiple rate PTP (mPTP) (Kapli et al., 2017); and Automatic Barcode Gap Discovery (ABGD) (Puillandre et al., 2012). Ultrametric trees generated from *cytB* and *coi* data of the Bayesian phylogeny were used as an input file for sGMYC and mGMYC. Previous studies have shown that the clock model and tree prior have low impact on the results of both sGMYC and mGMYC (Tavalera et al., 2013). These two analyses were conducted in R (R Core Team, 2017), using the package SPLITS (Ezard et al., 2009). We re-ran the phylogenetic reconstructions under the same parameters used in BEAST using MrBayes v. 3.2.6 (Huelsenbeck and Ronquist, 2001) to generate a bifurcating phylogram (with branch length representing the number of substitutions) to be the input file for PTPs analyses. bPTP analyses were performed in the online server^2^. The analysis length was 500,000 generations with sampling of 500 and burn-in of 0.1. Convergence was visualized on the MCMC interactions plots vs. log-likelihood. mPTP was run using the online server^3^, under the same parameters as for bPTP. ABGD distance-based analyses were run through the software command line, with a gap width value of 1.0 for all the distances available (*p*-distance, K2P, and Jukes–Cantor). The ABGD delineation taken into account was the one with the *P*-value of ∼0.01, as advocated by previous studies (Puillandre et al., 2012; Blair and Bryson, 2017). To have a species-delimitation partition based on a genetic distance threshold value, we used cut-off values of 5% divergence for *cytB* and 2% divergence for *coi* as indicators of distinct species. These values were based on previous reviews on genetic distances on fish species (Ward, 2009; Kartavtsev, 2011).

## Results

A total of 10 *cytB* and six *coi* haplotypes were found, none of which were shared among localities. For both mtDNA markers, the haplotype networks showed multiple mutational steps among all haplotypes (**Figure 1**). Mutations were especially abundant along the branch connecting KAE and the other populations (**Figure 1**). Genetic *p*-distances and K2P distances are presented in **Table 2**. Between KAE and the other localities, divergence values ranged from 7.5 to 8.0% for *cytB* and 4.9 to 5.0% for *coi*; between SJO and rivers that flow to the Guanabara Bay (GAC, GMI, and SAL) from 3.6 to 4.4% (*cytB*) and 2.2% (*coi*); and, among the watersheds that run into Guanabara Bay, from 2.6 to 3.2% (*cytB*) and 1.4% (*coi*) (**Table 2**).

**Table 2:** *P*-distances of cytochrome b gene (below diagonal) and K2P distances of cytochrome oxidase subunit I gene (above diagonal) for *Pareiorhaphis garbei* in the Serra do Mar coastal basins, Rio de Janeiro State, southeastern Brazil.

	KAE	SJO	GAC	GMI	SAL
Macaé (KAE)		0.049	0.050	0.050	–
São João (SJO)	0.080		0.022	0.022	–
Guapiaçu (GAC)	0.080	0.037		0.014	–
Guapimirim (GMI)	0.080	0.044	0.032		–
Santo Aleixo (SAL)	0.075	0.036	0.028	0.026	

**FIGURE 2:**
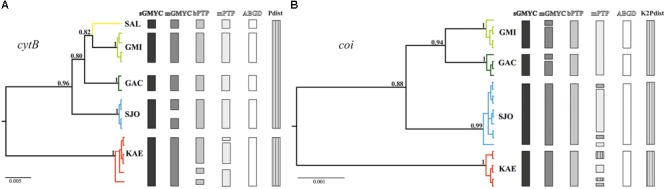
Ultrametric trees generated by the Bayesian phylogenetic reconstructions for **(A)** cytochrome b (*cytB*) and **(B)** cytochrome oxidase I (*coi*) in *Pareiorhaphis garbei*. The bars in different colors and patterns represent the operational taxonomic units (OTUs) partitions obtained by the different species delimitation methods.

Our phylogenetic reconstructions showed high genetic structuring within *P. garbei*, with all sampling sites representing monophyletic clusters in both the *cytB* and the *coi* data. According to this result, SJO is more closely related to the Guanabara Bay populations (GAC, GMI, and SAL) than to the KAE. Within the Guanabara Bay watersheds, GMI is more closely related to SAL (from an adjacent basin) than it is to GAC (that belongs to the same drainage, Guapi-Macacu), although supported by lower posterior probabilities (**Figure 2**).

The sGMYC and ABGD delimitation analyses indicated five distinct OTUs, corresponding to each sampling site for *cytB*. mGMYC split the SJO clade into two different OTUs, resulting in six genetic clusters. Both PTP methods (bPTP and mPTP) indicated potential distinct lineages within the KAE clade. The strict species threshold (*p*-distance > 5%) separates KAE from the other sampling sites (**Figure 2A**). Regarding to the *coi* reconstruction, four OTUs corresponding to sampling sites were shown as the best genetic partition in three of the five species-delineation methods (sGMYC, bPTP, and ABGD). However, some incongruences were found. Similarly to the *cytB* reconstruction, mGMYC tended to split lineages, dividing the GAC clade into two different clusters. Furthermore, mPTP found putative distinct genetic lineages within the SJO and KAE clades, resulting in a total of seven OTUs. Using the K2P distance species threshold value (>2%), the Guanabara Bay drainages represent one species while SJO and KAE each represent different putative species (**Figure 2B**).

Our paleo-drainage reconstruction places Macaé (KAE) and São João (SJO) in the same paleo-drainage (the Macaé-São João paleo-river), whereas Guapiaçu (GAC), Guapimirim (GMI), and Santo Aleixo (SAL) all reside in the Guanabara paleo-river (**Figure 1A**). This paleo-reconstruction does not corroborate the phylogenetic result, according to which SJO is somewhat more closely related to Guanabara Bay rivers populations than to KAE.

## Discussion

Our results revealed a discordance between past paleo-river connections and current genetic structure, which might indicate an ancient stream capture event in the headwaters of the Serra do Mar mountains. According to the paleo-drainage scenario, it was expected that SJO would be closely related to KAE; however, it appears to be a sister group to lineages from the Guanabara paleo-river. This result corroborates the river-capture event suggested by Maia et al. (2013) based on the occurrence of *P. garbei* in a single tributary adjacent to the headwaters of Macaé river basin. These drainage rearrangements are the result of tectonic reactivation that started in the Paleogene and continues to the present (Ribeiro et al., 2006; Lima and Ribeiro, 2011; Lima et al., 2016).

Although located within the Guapi-Macacu river basin, the water path connecting GAC and GMI is a lowland area where micro- and meso-habitats suitable for *P. garbei* are absent. The fact that GAC and GMI share no haplotypes, and belong to distinct lineages, reveals that any dispersal of *P. garbei* through the lower portions of rivers is very limited (and might explain in part why the current genetic structure of *P. garbei* does not perfectly reflect the paleo-drainage reconstruction).

Along the geographic range of *P. garbei*, a geographic barrier known as the Cabo Frio Magmatic Lineament (CFML) (Riccomini et al., 2005) may have influenced the evolution and diversification of many freshwater fishes. Based on molecular data, Pereira T.L. et al. (2013) suggested that this barrier had a vicariant effect isolating an eastern lineage (including a clade in the São João and Macaé basins) from a southeastern lineage (from Guanabara Bay to Paranaguá Bay) in the trahira, *Hoplias malabaricus* (Bloch, 1794). This pattern is also corroborated by the geographic distribution of *Atlantirivulus* rivulids in lowland areas (Costa, 2014). Although the CFLM seems to be an effective barrier to *P. garbei* (because different lineages are on its alternate sides), specimens from the São João basin are more closely related to Guanabara Bay drainages than to Macaé drainage, suggesting that different biogeographic forces may apply to headwater versus lowland fishes. Although not exclusively a headwater species, a biogeographic pattern similar to that found in *P. garbei* is also shown by *Hisonotus* loricariids, with *H. notatus* Eigenmann and Eigenmann, 1889 occurring from the São João river basin to the south (including Guanabara Bay’s drainages), whereas *H. thayeri* Martins and Langeani, 2016 occurs from the Macaé river northward (Martins and Langeani, 2016). Altogether, these various patterns indicate a complex and dynamic history of the coastal basins of the Fluminense ecoregion. Curiously, the CFLM is also the boundary between two marine provinces: the Tropical Southwestern Atlantic and the Warm Temperate Southwestern Atlantic (Spalding et al., 2007).

*Pareiorhaphis garbei* was the subject of a taxonomic review (Pereira and Reis, 2002; Pereira, 2005) that did not include molecular data, preventing the discovery of deep genetic structure. Several other demersal Neotropical freshwater fishes that display only subtle morphological differences have proved to show substantial genetic divergences that support the description of new and often endemic species (Benine et al., 2009; Melo et al., 2011; Cherobim et al., 2016), and some of these support an ancient split between rivers flowing to the Guanabara Bay versus the São João and Macaé drainages (Villa-Verde et al., 2012; Costa, 2014; Roxo et al., 2017). All molecular analyses herein performed indicate high divergence of the KAE population of *P. garbei* from all others, strongly suggesting that individuals from these two groups represent different species. If this outcome is formally recognized, *P. garbei* (*sensu stricto*) would be confined to the Macaé river basin (its type locality), with another species distributed along the headwaters of São João, Guapi-Macacu, and Santo Aleixo river basins. Moreover, according to high divergence at *coi*, the São João lineage could also be a distinct species from the one inhabiting drainages entering Guanabara Bay. In either case, *P. garbei* itself would be limited to Macaé river basin, in which the invasive rainbow trout poses a risk factor (Lazzarotto et al., 2007; Pereira and Brito, 2008). Currently, *P. garbei* is classified as ‘near threatened’ [Instituto Chico Mendes de Conservação da Biodiversidade [ICMBio], 2014], a category that probably stems from the recent record of this species in the São João river and the confirmation of its occurrence in protected areas (Maia et al., 2013). However, if the deep phylogenetic lineages found here corroborate a species complex in *P*. *garbei*, the conservation status of the entire assemblage may need reexamination, mainly due to a reduction in the range of each species due to the taxonomic splitting.

In summary, our molecular analysis together with paleo-drainage reconstructions revealed that the current phylogeographic patterns of the rheophilic catfish *P. garbei* were importantly impacted by headwater stream-captures. This taxon proved to encompass at least two highly divergent lineages, and furthermore, each headwater seems to represent a genetically diagnosable OTU. The integrative approach employed here helps to introduce a useful way to test hypotheses regarding to the distribution and conservation of Neotropical freshwater fishes.

## Author Contributions

SL had substantial contribution to the acquisition of samples, analyses, and interpretation of the results, writing the manuscript. WB-F and TA also contributed in the analyses, writing, and figures. HL helped in the fish collection, design, and writing the manuscript. AT and JA provided logistic support for molecular data during SL’s postdoctoral activities at UCI. All authors contributed in conception and elaboration of the manuscript, and read and approved its final version.

## Conflict of Interest Statement

The authors declare that the research was conducted in the absence of any commercial or financial relationships that could be construed as a potential conflict of interest.

## Footnotes

1 http://www.gebco.net/

2 http://species.h-its.org

3 http://mptp.h-its.org

## Supplementary Material

FIGURE S1 Bayesian phylogenetic reconstructions for cytochrome b (*cytB*) and cytochrome oxidase I (*coi*) in *Pareiorhaphis garbei*, using HKY+I (*cytB*) and TrN+G (*coi*) as nucleotide substitution models. *Pareiorhaphis* cf. *bahianus* (MG496258 and MG496259) from Contas river basin (northeastern Brazil) was used as out-group. Posterior probabilities are shown above the nodes.
