# Supplementary figures and images for: Headwater Capture Evidenced by Paleo-Rivers Reconstruction and Population Genetic Structure of the Armored Catfish (Pareiorhaphis garbei) in the Serra do Mar Mountains of Southeastern Brazil
Source: Front Genet. 2017 Dec 5;8:199. doi: 10.3389/fgene.2017.00199 (PMC5723395; doi:10.3389/fgene.2017.00199)

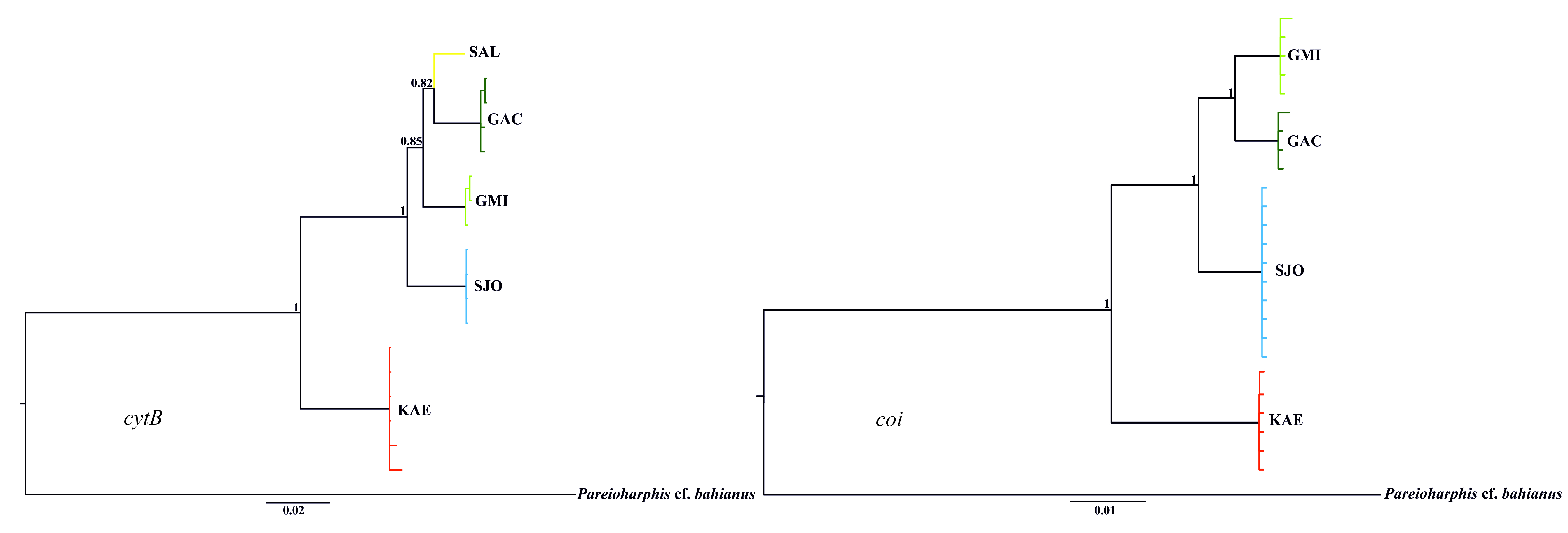

Supplement: FIGURE S1 — Bayesian phylogenetic reconstructions for cytochrome b (cytB) and cytochrome oxidase I (coi) in Pareiorhaphis garbei, using HKY+I (cytB) and TrN+G (coi) as nucleotide substitution models. Pareiorhaphis cf. bahianus (MG496258 and MG496259) from Contas river basin (northeastern Brazil) was used as out-group. Posterior probabilities are shown above the nodes. [file Image_1.jpeg]
